# Profile of illness in Syrian refugees: A GeoSentinel analysis, 2013 to 2015

**DOI:** 10.2807/1560-7917.ES.2016.21.10.30160

**Published:** 2016

**Authors:** FP Mockenhaupt, KA Barbre, M Jensenius, CS Larsen, ED Barnett, W Stauffer, C Rothe, H Asgeirsson, DH Hamer, DH Esposito, P Gautret, P Schlagenhauf

**Affiliations:** 1. Institute of Tropical Medicine and International Health, Charité – Universitätsmedizin Berlin, Berlin, Germany; 2. Division of Global Migration and Quarantine, National Center for Emerging and Zoonotic Infectious Disease, Centers for Disease Control and Prevention, Atlanta, United States; 3. Department of Infectious Diseases, Oslo University Hospital, Oslo, Norway; 4. Department of Infectious Diseases, Aarhus University Hospital, Skejby, Aarhus, Denmark; 5. Maxwell Finland Laboratory for Infectious Diseases, Boston Medical Center, Boston, United States; 6. Division of Infectious Diseases and International Medicine, University of Minnesota Medical School, St Paul, United States; 7. University Medical Center Hamburg-Eppendorf, Department of Tropical Medicine and Infectious Diseases, Bernhard Nocht Clinic, Hamburg, Germany; 8. Department of Infectious Diseases, Karolinska University Hospital, Stockholm, Sweden; 9. Department of Global Health and Center for Global Health and Development, Boston University School of Public Health; Section of Infectious Diseases, Department of Medicine, Boston Medical Center, MA, USA; 10. University Hospital Institute for Infectious and Tropical Diseases, Aix-Marseille University, Marseille, France; 11. University of Zürich Centre for Travel Medicine, WHO Collaborating Centre for Travellers’ Health, Epidemiology, Biostatistics and Prevention Institute, Zürich, Switzerland

## Abstract

Screening of 488 Syrian unaccompanied minor refugees (< 18 years-old) in Berlin showed low prevalence of intestinal parasites (*Giardia*, 7%), positive schistosomiasis serology (1.4%) and absence of hepatitis B. Among 44 ill adult Syrian refugees examined at GeoSentinel clinics worldwide, cutaneous leishmaniasis affected one in three patients; other noteworthy infections were active tuberculosis (11%) and chronic hepatitis B or C (9%). These data can contribute to evidence-based guidelines for infectious disease screening of Syrian refugees.

By the beginning of 2016, more than 4.6 million Syrians had crossed international borders since the civil war began in Syria in 2011. Most of these refugees are currently in Turkey (> 2 million), as well as in Lebanon, Jordan and Iraq. More than 800,000 asylum applications have been filed in Europe [1], and an unknown number of refugees in Europe have not yet been registered.

Access to healthcare is an important part of the human-itarian response to this crisis. To date, there is a lack of epidemiological or clinical data that can be used to guide screening for the most prevalent health conditions in this large refugee population. The goal of this report is to present the results of screening of a cohort of unaccompanied Syrian minors (UAMs) at the Berlin GeoSentinel site and to list some of the specific infectious diseases diagnosed among Syrian refugees who presented at GeoSentinel sites worldwide.

## Inclusion criteria and analytical methods

Patient records were drawn from the GeoSentinel Surveillance System. This is a clinic-based global surveillance network of 63 travel and tropical medicine clinics. To be eligible for inclusion in the database, the patient must have crossed an international border before presentation and the diagnosis (556 possible diagnostic codes) must be considered to be travelrelated. Other data captured include demographic information (age, sex, country of birth, country of residence and citizenship), travel history, reason for travel and possible area of illness acquisition [2].

Two groups were analysed. Group 1: A cohort of UAMs younger than 18 years screened for infectious diseases (except tuberculosis) at the Berlin GeoSentinel site as part of routine UAM arrival procedures in the city. Group 2: Patients who presented to GeoSentinel sites worldwide and who were diagnosed with a confirmed or probable illness related to migration. In both groups, analysis was limited to migrants who reported birth or residence before the age of 10 years in Syria, who arrived in their present country of residence in March 2011 or later and who presented to a GeoSentinel site before 1 December 2015. Data on date of departure from Syria were not collected. In Group 1, approval for participation in the GeoSentinel surveillance was provided by the legal representative of the UAMs (Berlin Senate Department for Education, Youth and Science) and ethical clearance was provided by the Ethics Committee of Charité – Universitätsmedizin Berlin. For Group 2, data collection among adult patients represents public health surveillance. All UAMs were screened for intestinal parasites (microscopy, immunofluorescence) and had serology testing for schistosomiasis and hepatitis B (anti-HbS, anti-Hbc, HbS antigen). Further laboratory tests were done based on medical discretion. Screening for pulmonary tuberculosis was performed elsewhere and these data were not available to us. Standardised psychological assessments were not performed. All analyses were conducted with SAS 9.3.

## Results

### Group 1: Screened unaccompanied minors

A total of 488 UAMs were screened at the Berlin site from October 2013 through November 2015. The majority were male (94%), aged 16 to 17 years (64%), Syrian-born (99%) and evaluated within 42 days of arrival in Germany (62%) (Table 1).

UAMs reported up to seven transit countries, the most frequently named being Turkey, Greece, Serbia, the Former Yugoslav Republic of Macedonia and Hungary (Table 1). Results of the screening and examinations performed revealed no infections or clinically overt disease in two thirds of the UAMs (Table 2).

Twenty-two per cent of the UAMs were diagnosed with at least one intestinal parasite, including *Giardia duodenalis* (7%), *Blastocystis* sp. (12%) and other non-pathogenic protozoa (6%). Serology for schistosomiasis was positive in seven (1.4%) UAMs (without excretion of eggs). None tested positive for hepatitis B.

### Group 2: Syrian migrants diagnosed at GeoSentinel sites

The analysis of other Syrian migrants diagnosed at GeoSentinel clinics worldwide included 44 patients evaluated in eight countries between June 2011 and November 2015. The majority of these were male (n = 29) and Syrian-born (n = 43) (Table 3).

The median age was 35 years (range: 1–67). The most frequent diagnoses in this group included: cutaneous leishmaniasis (n = 14), active (n = 5) and latent (n = 4) tuberculosis and chronic hepatitis (B or C, n = 4).

## Discussion

Our analysis indicates that the majority of predominatly male Syrian UAMs presenting in Berlin from October 2013 through November 2015 posed very limited infectious risk. Screening of the UAMs showed mostly intestinal parasites (22%) and positive schistosomiasis serology (1.4%). The evaluation of a small number of adult Syrian migrants of which two thirds were men and diagnosed at GeoSentinel sites with illnesses related to migration, probably acquired before departure from Syria, showed that cutaneous leishmaniasis, tuberculosis and chronic hepatitis may be encountered in this population.

Early in the refugee crisis, increased rates of leishmaniasis and tuberculosis were observed among Syrian refugees in neighbouring countries [3,4]. Recent data on Syrian refugees in Jordan show a prevalence of 158/100,000 for cutaneous leishmaniasis, 13/100,000 for tuberculosis and 51/100,000 for measles [5,6]. In Lebanon in 2013, 47% of Syrian patients had skin diseases (cutaneous leishmaniasis, scabies, lice, staphylococcal infection) and 2% had systemic infectious diseases (measles, hepatitis, typhoid fever) [7]. There, 1,033 new cases of leishmaniasis (99.8% cutaneous) were reported in 2013, virtually all in Syrian refugees, compared with between none and six cases in previous years [8]. Cutaneous leishmaniasis has also been reported in refugees in Turkey [9,10], and the recent emergence of *Leishmania major* and *L. donovani* has been attributed to the influx from Syria [11,12].

In contrast, hardly any data are available regarding the health of Syrian refugees arriving in the European Union. Reassuringly, no importation of wild-type poliovirus was detected among 629 Syrian refugees of toddler age in Germany [13]. Although most UAMs screened free of infectious disease, 7% had *G. duodenalis* infection, which could lead to further transmission (e. g. under crowded conditions and considering the sometimes substantial delay until screening). On the other hand, this figure is only slightly higher than the proportion of giardiasis in international travellers returning to Europe [14] and it accords with the comparatively low prevalence of parasitic diseases observed in a small group of UAMs from western Asia (Syria, Iraq, Georgia) arriving in Germany in 2011 to 2014 [15].

Among adult Group 2 refugees, we detected five cases of tuberculosis disease. Despite the limitations of small group size and lacking denominator, this accords with a recent World Health Organization classification of Syria as a low-incidence tuberculosis country. However, clinicians treating Syrian patients should consider multidrug-resistant tuberculosis (6% and 31% in new and retreatment cases, respectively) [16]. In addition, we detected 14 cases of cutaneous leishmaniasis among adult Syrian migrants. Although based on few numbers, this finding together with published work [3–10] confirm that cutaneous leishmaniasis is encountered in this population. This warrants increased awareness of the condition among healthcare professionals treating Syrian refugees.

One limitation of this study is that data on the UAMs were influenced by issues of translation and comprehension, as well as reluctance to disclose sensitive information. Inconsistencies were also observed with respect to the stated travel routes. In addition, depressive and post-traumatic stress disorders were not systematically assessed in the present study but were reported among 20% of UAMs from western Asia in a previous study [15].

Current European Union-wide regulatory frameworks and screening guidelines [17,18] do not specifically address Syrian refugees. Our data have public health implications in that they augment the very limited evidence base that is available to formulate screening guidelines for infectious diseases in Syrian refugees arriving in Europe. The results suggest that young refugees from Syria have a low prevalence of potentially harmful parasite infection such as *Giardia* and schistosomiasis, but these two should be included in screening protocols. Poor hygiene facilities at refugee centers may increase the transmission of *Giardia* and of other intestinal pathogens. Improving hygiene conditions, more rapid screening and (presumptive) treatment are possible countermeasures. Although the UAMs did not undergo psychological assessment or counselling, based on clinical impression, such is imperative. Syrian adults, in this study based on very small numbers, presented with cutaneous leishmaniasis, tuberculosis, and hepatitis B indicating that screening protocols for adults should address these infections and that resources need to be assigned for screening, treatment and follow-up. The Syrian refugee crisis necessitates targeted action on infectious disease, mental health and chronic illness [19] and intensive collaboration of all public health partners involved in refugee care.

## Figures and Tables

**Table 1:** Demographic information for unaccompanied minors screened at the Berlin GeoSentinel site after migration from Syria, October 2013–November 2015 (n = 488)

Characteristic	Number	Percentage
Male sex	458	94
Age (years)		
6–9	8	2
10–12	34	7
13–15	136	28
16–17	310	64
Born in Syria^a^	485	99
Time elapsed between arrival and evaluation (days)		
0–14	54	11
15–28	146	30
29–42	102	21
43–56	53	11
57–70	35	7
> 70	64	13
Missing arrival date	34	7
Number of transit countries		
1	48	10
2	69	14
3	41	8
4	42	9
5	69	14
6	58	12
7	21	4
None specified	140	29
Specific transit countries^b^		
Turkey	296	85
Greece	217	62
Serbia	172	49
Former Yugoslav Republic of Macedonia	149	43
Hungary	145	42
Austria	97	28
Lebanon	59	17
Italy	58	17
Egypt	23	7
Libyan Arab Jamahiriya	21	6
Algeria	12	3
Jordan	10	3

a Three minors born outside of Syria reported birth countries of Libyan Arab Jamahiriya, Palestinian Territory and Saudi Arabia. All were older than 10 years and reported residence in Syria before age 10 years.

b Percentages refer to 349 minors with available travel history. The Table is limited to countries reported by 10 or more patients. Additional countries included: Croatia (n = 8 patients), Bulgaria (n = 7), France (n = 7), Tunisia (n = 6), Cyprus (n = 4), Spain (n = 4), Morocco (n = 3), Slovenia (n = 3), Sudan (n = 3), Albania (n = 2), Australia (n = 2), Czech Republic (n = 1), Iran (n = 1), Iraq (n = 1), Malta (n = 1), Montenegro (n = 1), the Netherlands (n = 1), Qatar (n = 1), Russian Federation (n = 1) and Sweden (n = 1).

**Table 2:** Diagnosis information for unaccompanied minors screened at the Berlin GeoSentinel site after migration from Syria, October 2013–November 2015 (n = 488)^a^

Diagnosis	Number	Percentage^b^
None	324	66
At least one intestinal parasite infection^c^	108	22
Blastocystis	58	12
Giardia	34	7
Other non-pathogenic protozoa	27	6
Unspecified intestinal parasite	4	< 1
Eosinophilia	17	3
Abnormal urinalysis	7	1
Anaemia	7	1
Schistosomiasis (any species)	7	1
Dental problems	5	1
Fungal infections	5	1
Scabies	3	< 1
Upper respiratory tract infection	2	< 1

a This table includes diagnoses affecting two or more minors. Additional diagnoses affecting one each included: abdominal pain of unspecified aetiology, arthralgia/bone pain, acute bronchitis, chronic brucellosis, cough of no aetiology, acute unspecified diarrhoea, hookworm, influenza-like illness, other intestinal parasite, laryngitis, leukopenia, poor vision/vision loss, intestinal strongyloidiasis, syncope, trichuriasis, non-genital warts and weight loss.

b 26 patients had more than one recorded diagnosis. This included 23 patients with two diagnoses, one with three diagnoses, one with four diagnoses and one with five diagnoses.

c 15 patients were diagnosed with more than one intestinal parasite. This included 14 patients diagnosed with two parasites and one patient diagnosed with three parasites.

**Table 3:** Demographic and diagnosis information for patients presenting at GeoSentinel sites after migration from Syria, June 2011–November 2015 (n = 44)^a^

Characteristic	Number
Male sex	29
Age (years)	
> 18	13
18–30	9
31–50	18
51–67	4
Born in Syria^b^	43
GeoSentinel site country	
Norway	15
United States	9
Denmark	7
Canada	6
Germany	4
France	1
Sweden	1
Switzerland	1
Diagnosis^c^	
Cutaneous leishmaniasis	14
Active tuberculosis	5
Pulmonary	3
Extrapulmonary	2
Chronic hepatitis	4
Hepatitis B	3
Hepatitis C	1
Latent tuberculosis	4
Vitamin D insufficiency	4
Dental problems	3
Nonseptic arthritis	2
Antibiotic-resistant pyelonephiritis	2

a Includes patients who received at least one final, probable or confirmed diagnosis and excludes patients between the ages of six and 17 years evaluated at the Berlin GeoSentinel site.

b One patient was born in Somalia, but lived in Syria before the age of 10 years.

c Two patients had more than one recorded diagnosis. Both of these patients had three diagnoses each. The table reflects diagnoses affecting two or more patients. Additional diagnoses affecting one patient each included abnormal urinalysis, arthralgia, blastocystosis, constipation, hepatic echinococcosis, enterobiasis, angina, hypertension, nonpathogenic protozoa (other than *Blastocystis*) and post-traumatic stress disorder.
